# Enhanced Antimicrobial Activity through Synergistic Effects of Cold Atmospheric Plasma and Plant Secondary Metabolites: Opportunities and Challenges

**DOI:** 10.3390/molecules28227481

**Published:** 2023-11-08

**Authors:** Karthika Prasad, Syamlal Sasi, Janith Weerasinghe, Igor Levchenko, Kateryna Bazaka

**Affiliations:** 1 School of Engineering, College of Engineering, Computing and Cybernetics, The Australian National University, Canberra, ACT 2600, Australia; syamlal.sasi@anu.edu.au (S.S.); janith.adikarammudiyanselage@anu.edu.au (J.W.); levchenko.igor@nie.edu.sg (I.L.); 2 Plasma Sources and Application Centre, NIE, Nanyang Technological University, Singapore 637616, Singapore

**Keywords:** antibiotic resistance, cold atmospheric plasma, secondary metabolites

## Abstract

The emergence of antibiotic resistant microorganisms possesses a great threat to human health and the environment. Considering the exponential increase in the spread of antibiotic resistant microorganisms, it would be prudent to consider the use of alternative antimicrobial agents or therapies. Only a sustainable, sustained, determined, and coordinated international effort will provide the solutions needed for the future. Plant secondary metabolites show bactericidal and bacteriostatic activity similar to that of conventional antibiotics. However, to effectively eliminate infection, secondary metabolites may need to be activated by heat treatment or combined with other therapies. Cold atmospheric plasma therapy is yet another novel approach that has proven antimicrobial effects. In this review, we explore the physiochemical mechanisms that may give rise to the improved antimicrobial activity of secondary metabolites when combined with cold atmospheric plasma therapy.

## 1. Introduction

Antibiotics, containing unique physicochemical properties, can hinder bacterial overgrowth through various mechanisms, including but not limited to direct bacterial destruction or inhibition of protein synthesis, which in turn hinders their growth and replication [1,2]. The discovery of modern antibiotics was a major breakthrough in medicine and has saved countless lives by effectively treating bacterial infections that were once deadly [3]. However, the overuse and misuse of antibiotics has led to the development of antibiotic-resistant bacteria, which is a growing public health concern as the number of difficult to treat infections is increasing, and new antibiotics are not being developed at the same pace [4]. Antibiotic resistance can emerge due to a variety of factors, including the inappropriate and indiscriminate use of antibiotics in humans, animals, aquaculture and agriculture, as well as the spread of resistant bacteria through person-to-person contact due to the unprecedented levels of global mobility, contaminated food and water, and other sources [1,5].

Antibiotic resistant bacteria evolve and develop a broad range of mechanisms that enable them to resist the effects of the drugs, allowing them to multiply and spread uncontrollably under conditions that would significantly limit the growth of susceptible strains. The development of antibiotic-resistant strains that are difficult or impossible to treat with existing antibiotics and other traditional treatments has serious implications for public health, as well as for individual patients who may be left with limited options for treatment [1,5,6]. The ever-increasing rate with which antibiotic-resistant strains of bacteria continue to emerge highlights the need for increased efforts to preserve the effectiveness of antibiotics and to develop new treatments/alternative therapies that can effectively target these resistant strains.

Plant secondary metabolites (PSMs), which are biologically active compounds produced by plants, have gained attention as alternative therapies for various medical conditions [7,8]. These compounds have been shown to have a wide range of pharmacological activities, including anti-inflammatory, antioxidant, and antimicrobial properties [9,10]. They are often used in traditional medicine and are being studied for their potential to treat a variety of health problems, such as infections, inflammation, cancer, and cardiovascular disease [11]. However, the use of secondary metabolites alone cannot replace antibiotics.

Cold atmospheric plasma (CAP) is a non-thermal technology that is attracting interest as an alternative therapy for various medical conditions [12,13,14]. CAP is typically generated by passing a gas through an electrical discharge, creating a plasma that contains reactive species such as reactive oxygen and nitrogen species (ROS and RNS), ions, electrons, and UV photons [15,16]. These species can interact with biological materials and have been shown to have a wide range of therapeutic effects, including antimicrobial, anti-inflammatory, and wound-healing properties [15]. CAP has been shown to be effective against various microorganisms, including bacteria, viruses, and fungi, and has been investigated as a potential treatment for infections and other conditions, such as cancer and dermatological disorders [17,18]. While CAP has shown promise as an alternative therapy, more research is needed to fully understand its mechanisms of action and to determine the most effective doses and delivery methods [19]. Despite these uncertainties, CAP is gaining attention as a potential new therapy for various medical conditions [15,20].

In this review article, we provide a comprehensive overview of the mechanism of antibiotic resistance along with insights into alternative therapies for treating bacterial infections such as the use of secondary metabolites and cold atmospheric plasma therapy.

## 2. Mechanisms of Antibiotic Resistance

The overuse of antibiotics is a growing concern and has serious consequences for public health such as the development of antibiotic resistant bacterial strains [21]. Antibiotic resistance refers to the ability of bacteria to survive and continue to multiply even in the presence of antibiotics. Bacteria can develop an ability to resist antibiotics through several mechanisms, including altering the structure of the antibiotic target, reducing the uptake of the antibiotic into the bacterium, or by producing enzymes that inactivate the antibiotic [22,23]. Persistence refers to the ability of a small subset of bacteria to survive exposure to antibiotics but not multiply [24]; these bacteria are not killed by the antibiotics but instead enter a dormant state called a persistent phenotype and can be reawakened later when the antibiotic pressure is reduced. This is a survival mechanism that allows bacteria to persist in the presence of antibiotics and can contribute to the development of chronic infections and antibiotic resistance [25,26]. Both persistence and resistance can contribute to the failure of antibiotics to cure infections and the spread of antibiotic-resistant bacteria [27].

**Figure 1:**
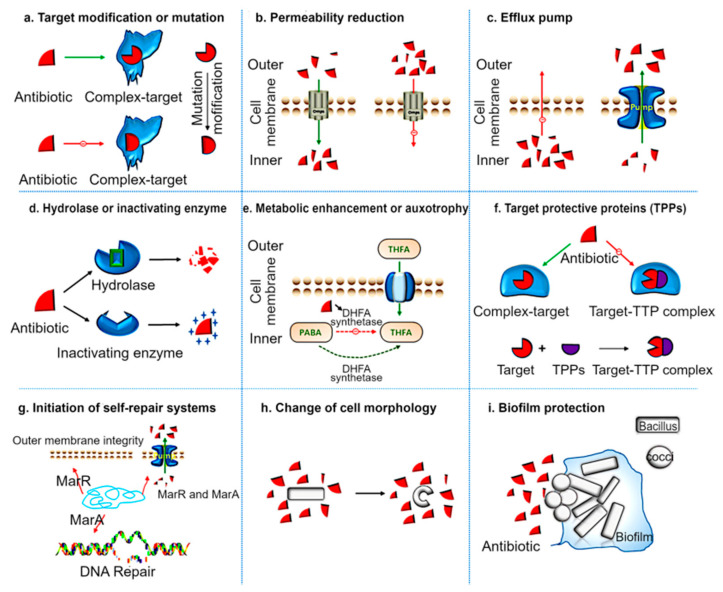
Different mechanisms involved in antibiotic resilience (**a**) Modification of the target preventing the antibiotics from combining with the target. (**b**) Reducing the permeability of cell membrane to reduce the intake of antibiotics. (**c**) Expulsion of the drugs from cells through efflux pumps. (**d**) Hydrolyze enzyme inactivating the antibiotics after entering the cell. (**e**) Metabolic enhancement to limit or alter the sensitivity of the bacteria towards antibiotics. (**f**) Developing target protective proteins which prevents the antibiotics from attaching to the bacterial target. (**g**) Initiation of self-repair system. (**h**) Changing the cell morphology to reduce the antibiotic update. (**i**) Formation of biofilms or existing as colonies to jointly resist the effect of antibiotics. Reproduced with permission from reference [28] © Zhang, F, et al. 2022 MDPI, Basel, Switzerland.

As explained in Figure 1, there are several mechanisms by with bacteria resist the effect of antibiotics or persist in their presence. Mutation, which can be acquired during the DNA replication process, is one of the common pathways by which bacteria gain and subsequently pass on the resistant genes from one generation to another. Mutation can help bacteria alter their cellular membranes, creating a modified target to which antibiotics cannot bind (Figure 1a) [28,29,30], change the properties of porin to reduce cell membrane permeability (Figure 1b) [31], modify efflux pumps that remove antibiotics out of the cell before the latter becomes activated (Figure 1c) [28,30], eventually develop antibiotic hydrolases or inactivating enzymes which can inactivate antibiotics after entering the cells (Figure 1d) [30,32], develop tolerance to antibiotics through enhancement in metabolic activities (Figure 1e) [33], develop protective proteins in the target site which inhibit the antibiotics from binding to the cell (Figure 1f) [30,34], modify the antibiotic resistant operon to initiate self-repair by restoring DNA and outer membrane integrity in gram-negative bacteria (Figure 1g) [30,35], and even alter cell morphology to reduce the area of exposure to antibiotics (Figure 1h) [30,36]. Biofilm formation is yet another effective way by which bacteria form resistance to antibiotics. In the natural environment, bacteria frequently exist in the form of multi-species communities, and this communal lifestyle can be highly effective in inhibiting the activity of antibiotics due to a combination of factors (Figure 1i) [30,37]. These include limited diffusion of antimicrobial agents through the biofilm matrix, communication of antimicrobial agents with the biofilm matrix and its constituent polymers and cells, enzyme-mediated resistance, varying levels of metabolic activity within the biofilm, genetic adaptation, efflux pumps, and outer membrane structures [38]. This resilience is also supported by the transition of biofilm colonies from exponential to slow or no growth states, known as persisters. The glycocalyx matrix, along with efflux systems and enzymes, plays a role in inactivating antimicrobial agents and shielding the outer regions of the biofilm. Interestingly, intermediate biofilm cells may experience nutrient starvation, slowing their growth. Changes in gene expression under stress conditions help protect surface-bound bacteria from cellular damage. Investigating the mechanisms of biofilm resistance, particularly by isolating antibiotic-resistant bacteria within biofilms, can enhance our understanding of these complex interactions, ultimately benefiting patient health and survival [39].

Biofilms’ antibiotic resistance mechanisms not only present a scientific challenge but also have significant effects for human health. These resistance factors pose challenges to effective antibiotic treatments, as they can render many traditional therapies less effective [40]. This is a matter of great concern, as infections associated with biofilms can be particularly stubborn and difficult to eliminate, leading to prolonged illness, increased healthcare costs, and in some cases, severe complications [41]. 

## 3. Possible Synergies between CAP and PSM Therapies

CAP is found to be effective against a wide range of muti-drug-resistant bacteria and biofilms [16,42,43,44]. There are several mechanisms by which CAP induces antimicrobial effect [45]. The UV radiation from CAP can destroy the microorganism by directly penetrating through the cell wall and reacting with intercellular components such as nucleic acids, leading to cell death. The charged particles present in the CAP can accumulate on the outer cell membrane and induce electrostatic stress, resulting in the rupture of the cell wall [46]. The reactive species, on the other hand, diffuse through the cell wall causing damage to the cytoplasmic membrane, microbial proteins, and DNA through oxidation [45,46]. 

Similarly, PSMs can alter the physiology, metabolism, and stress responses of microorganisms [47,48,49]. Exposure to PSMs can result in a reduction in enzymic activity, cause changes in DNA synthesis, and can also limit the metabolic activities of cells. At times, the PSMs can affect the sensing system within the bacterial community and thereby inhibit biofilm formation [50,51,52]. Importantly, many secondary metabolites generate ROS that can induce enzymatic oxidative stress responses in a bacterial cell [53,54]. 

**Figure 2:**
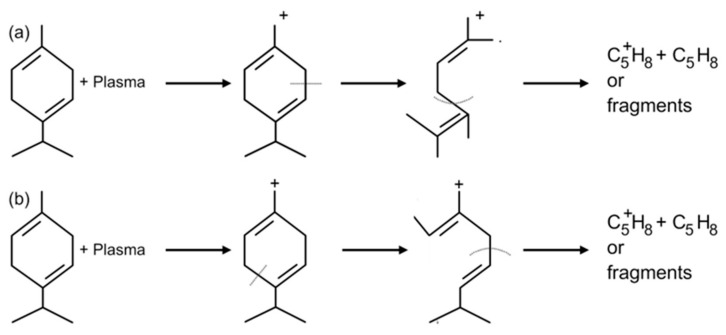
Schematic representation of possible structural changes induced by treating γ-terpinene with plasma. (**a**) represents a possible ring opening mechanism by fission, and (**b**) represents a possible cleavage at quaternary center. Reproduced with permission from [56] © 1999–2023 John Wiley&Sons.

Even though CAP and PSMs have different ways of killing microbes, the synergistic antimicrobial effect of CAP and PSMs is often linked to the modified chemical structure of PSMs post plasma treatment and the reactive species produced by CAP (Figure 2) [55]. Even though the mechanism behind the combined antimicrobial effect of CAP and PSM is not fully understood, here are some possible hypotheses: 

### 3.1. Change in Physiochemical Features of PSMs under Plasma

Plasma exposure has previously been shown to modify the chemical structure and composition of some secondary metabolites and their naturally occurring mixes (i.e., essential oils and extracts) through various reactions (Figure 2) [56]. It is found that active electrons in the CAP can cause fragmentation in the chemical structure of PSMs. CAP can alter the chemical structure of PSMs and create a mixture of chemically diverse species. It can even add biologically active functional groups, e.g., oxygen containing moieties that were not originally present in the PSM monomer [1,57]. These biologically active species can often have improved antimicrobial properties, which can enhance the antibacterial efficiency of PSMs. The nitrogen species produced by CAP can either react with the PSMs to form different compounds or can functionalize the surface of these molecules [58,59]. CAP can rearrange the functional groups or even change the functional groups present in the PSMs. CAP can also induce crosslinking reactions among different plant metabolites to promote the formation of new composites with enhanced antimicrobial properties [60,61]. The wide assortment of potentially active molecules can simultaneously target a variety of targets, limiting the ability of the cell to repair the damage and recover, and in doing so also minimizing the likelihood of resistance emerging.

### 3.2. Disruption of Cell Membrane

Similar to any effective antimicrobial agent, both CAP-generated active species and PSMs can cross the bacterial cell wall by disrupting the microbial cell membrane. With certain types of PSMs, it is the hydrophobic nature of the compound that allows it to accumulate on the cell surface, causing agitation in the structure and function of cell membrane [62]. Further interactions between PSMs and the bacterial cell wall can stress the biological function resulting in the failure of chemiosmotic control and making the membrane more permeable [62], whereas the combined impact of UV photons and reactive species found in CAP disrupts the microbial cell membrane through the generation of reactive oxygen and nitrogen species [63]. Therefore, it is hypothesized that the combination of the hydrophobic interactions and reactive species can induce irreversible damage to the cell membrane. 

### 3.3. Intercellular Damage Caused by Oxidative Stress 

**Figure 3:**
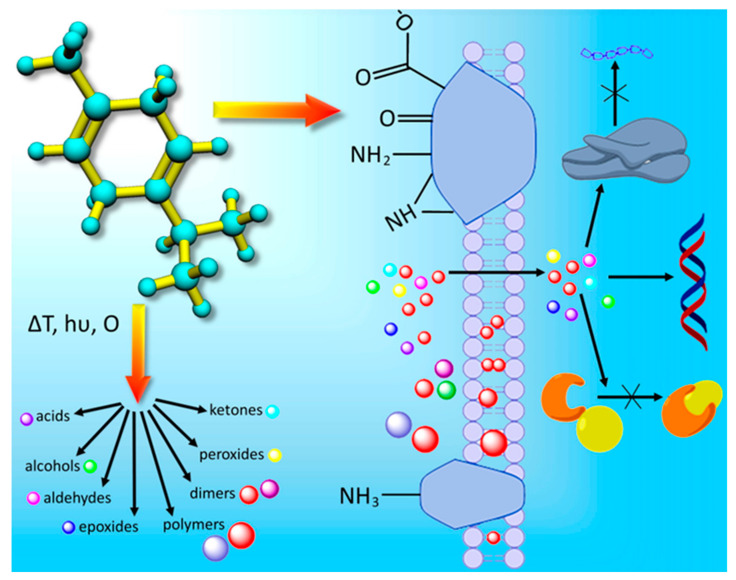
Potential interactions between terpenoid PSMs and bacterial cells may include the formation of hydrogen and ionic bonds with cell membrane proteins, disruption of membrane integrity, and intracellular interactions with cell components. Reproduced with permission from K. Bazaka et al., 2017 [1]. © IOP Publishing Ltd., 2017.

**Figure 4:**
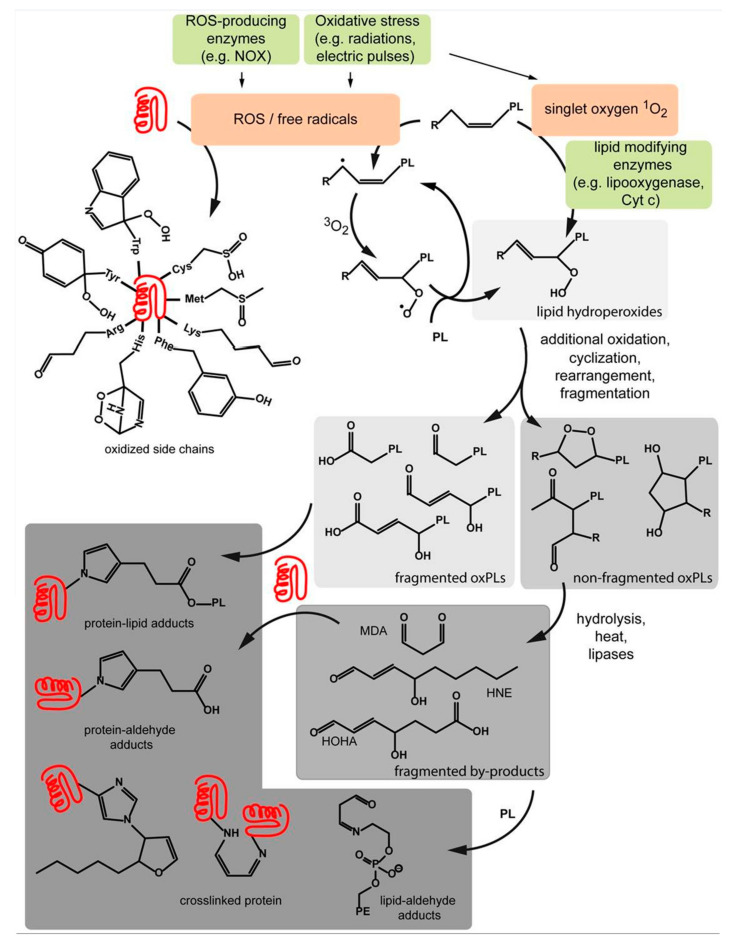
Schematic representation of the chemical process involved in the peroxide formation. ROS generation near the membrane can target the fatty acyl chain or the head group of phospholipids as well as the side chains of membrane proteins, resulting in the altered function of the bilayer. Reproduced with permission from reference [68] © 2017, American Chemical Society.

When there arises a difference between the production and composition of reactive oxygen species, the cells undergo oxidative stress, and microbes, especially bacteria, have a tendency to self-destruct under severe oxidative stress [64,65]. Both CAP and PSMs generate ROS that can produce a significant increase in oxidative stress experienced by the microorganism. PSMs can induce intercellular ROS production while forming complexes with intercellular proteins through hydrophobic interactions (Figure 3), while CAP generates a complex array of RONS, including hydroxyl radicals, singlet oxygen, superoxide radicals, and nitrogen oxides in gases through ionization reactions in liquids through the interactions between plasma-generated UV and gaseous species and molecules of liquids [1]. These RONS are crucial in the antimicrobial effects of CAP, as they induce oxidative stress, damage bacterial cell membranes, nucleic acids, and proteins, ultimately culminating in bacterial cell demise [66]. The increase in RONS can remove hydrogen from lipids and proteins of the microbial cell membrane, leading to the peroxidation of polyunsaturated fatty acids [67]. As shown in Figure 4, peroxide formation can eventually induce a chain reaction that spreads over to the other lipid bilayers, leading to the loss of cell membrane integrity [67,68]. The self-propagating peroxidization of unsaturated fatty acids can lead to the formation of lipid hydroperoxides, which can generate oxidized lipids such as malonyldialdehyde, hydroxynoneal, and 4-hydroxy-7-oxo-heptenoic acid through a process of repeated, truncated, or fragmentated oxidation. These oxidized products can then react with the cellular components too. In addition, it is also noticed that with CAP, the less hydrophilic species—specifically NO, NO_2_, N_2_O_4_, and O_3_—show greater permeability through lipid bilayers when compared to their hydrophilic counterparts, which include HNO_3_, s-cis-HONO, s-trans-HONO, H_2_O_2_, HO_2_, and OH [69].

In addition to the lipid bilayers, the RONS generated through the synergistic effect of CAP and PSMs can damage the nucleic acid of the DNA present inside the cell. RONS mostly react with DNA by causing significant oxidative reactions to the nitrogenous base and deoxy ribosome [67]. Oxidation can alter the DNA, break the double helix strand structure, and cause other mutagenic changes. Hydroxyl radicals, on the other hand, cause direct damage to the DNA by oxidization of pyrimidine and purine bases leading to the formation of 8-hydroxy-2-deoxygunoise and other oxidative lesions, which then interfere with usual DNA replication and transcription [70]. Similarly, mitochondria, the main source of cellular ROS, is also vulnerable to RONS-induced damage. RONS can disrupt the mitochondrial membrane, which can lead to the impairment of electron transport chain function, which can further lead to the overproduction of mitochondrial ROS and intensify the oxidative stress resulting in cell dysfunction or apoptosis [71,72]. 

Overall, the reactive species generated from CAP, along with the molecules in PSMs, alter various properties within the bacterial cell and lead to a series of reactions that results in the disruption of cell membrane integrity, the oxidization of lipids and proteins, mitochondrial and DNA damage, and activation of the pathways leading to cell death [68]. 

### 3.4. Transdermal Delivery

A number of in vitro, ex vivo, and in vivo studies have demonstrated that a brief exposure of skin to physical and chemical effects generated by CAP can lead to a transient enhancement in the efficacy of transdermal delivery of topically applied therapeutics, both small (~300–500 Da) and large (~3000–70,000 Da) [73,74,75], with results showing a substantial increase in the cumulative amount of drug being taken up and suggesting that a similar strategy can be used to improve the delivery of PSMs deeper into the skin (e.g., a wound) or even into systemic circulation. Importantly, molecules with large weight and size could be effectively diffused subsequent to CAP, overcoming one of the major challenges of using such therapeutics [76].

**Figure 5:**
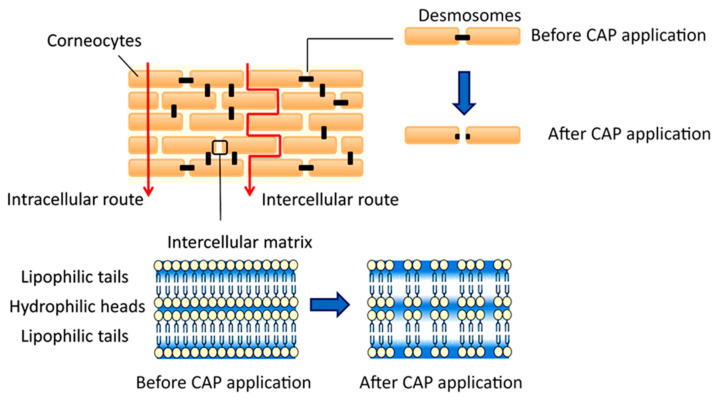
Mechanisms of plasma effect on skin permeability. CAP could temporarily downregulate the expression of E-cadherin, which plays a crucial role in the formation of the intracellular junctions. Loss of E-cadherin would damage the tight junctions between the keratinocytes and transiently disrupt the skin barrier function. Reprinted with permission from reference [85] © Controlled Release Society 2020. Published by Spinger.

The increase in permeability has been attributed to the significant and rapid increase in transepidermal water loss (TEWL) observed immediately post-exposure of skin to CAP. The increase was temporary in nature, with the TEWL indicator returning to its base value over time as the barrier function of the skin recovered [73]; the recovery time is dependent on the nature and the dose of CAP treatment [75]. Another measure of skin barrier function, stratum corneum hydration, also showed a reduction in its value, with the extent of the reduction depending on the treatment parameters and having a gradient across the depth profile of the skin layer [77]. Not surprisingly, given the high reactivity and short lifetime of plasma-generated species, the effects of CAP treatment were primarily localized to the topmost corneocyte layer [78]. Despite ample evidence of the improvement in transdermal delivery, the mechanisms by which CAP induces the observed change in skin permeability remains not fully understood. However, it has been speculated that the lipids within the stratum corneum layer, e.g., α-linolenic acid, can interact with ROS generated by CAP, e.g., •OH and singlet oxygen, producing oxidized fatty acid derivatives that render the matrix more hydrophilic and hence more permeable [79]. Extended exposure to CAP treatment may lead to lipid peroxidation and membrane poration [80,81]. In parallel to exposure to ROS and UV, certain types of CAP may generate substantial electric fields, which can result in transient intracellular and intercellular poration, possibly due to the downregulation of the expression of cell adhesion molecules such as E-cadherin and consequently greater movement of ions and molecules across the layer, which is lacking tight junctions that would otherwise connect keratinocytes (Figure 5) [82,83,84].

## 4. Potential Challenges of CAP-PSM Combination Therapy

In order to ensure optimal efficacy of the combination CAP-PSM therapy, it is important to explore whether there are mechanisms that may be antagonistic and may therefore lead to suboptimal therapeutic outcomes; as such, they may contribute to the emergence of resistant pathogens. Given that plants have evolved PSMs to, among other purposes, fight infectious agents, it is not surprising that microorganisms have evolved a wide range of defense strategies to protect themselves against PSM activity, and given that there is an overlap between how CAP and PSM target cells, exposure to PSMs may activate these defense mechanisms, thus possibly protecting the cell from the effects of CAP therapy. For instance, it has been suggested that the efflux systems in cells may have, in part, evolved to expel PSMs [86,87,88], and their activation would prevent CAP-modified PSMs from effectively entering the intracellular milieu. Furthermore, PSMs are likely to interact with ROS generated by CAP-generated and endogenous ROS, thereby potentially modulating the toxicity of the CAP treatment. Both mechanisms may become difficult to deconvolute, particularly when diverse PSMs are used, such as the case with the use of topical essential oils or plant extracts.

### 4.1. Efflux Systems

**Figure 6:**
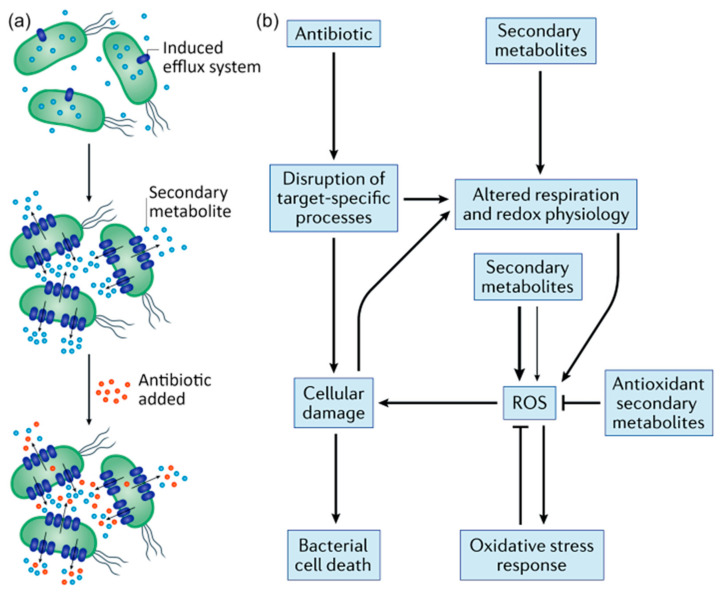
(**a**) Secondary metabolite-mediated regulation of multidrug resistance efflux pumps. Secondary metabolites induce the expression of efflux systems, which export the metabolite. The increased expression of efflux pumps can provide collateral resilience to antibiotics used in the clinic by expelling the drugs from the microbial cells. (**b**) Secondary metabolite interactions with oxidative stress. Schematic depicting how bactericidal antibiotics can cause cell death both by directly disrupting target-specific processes and by indirectly promoting the formation of reactive oxygen species (ROS) as a consequence of altered respiration and cellular damage. Secondary metabolites can interface with these pathways at multiple points, including by interfering with respiration and redox homeostasis, directly generating ROS through redox cycling and detoxifying ROS via one-electron reactions. Secondary metabolites that promote oxidative stress can either antagonize or potentiate antibiotic toxicity, which is likely to depend on whether the resulting increases in ROS are moderate (thin arrow) or severe (thick arrow). Moderate increases in ROS may induce protective oxidative stress responses that can counteract antibiotic toxicity, whereas severe increases in ROS may overwhelm the defenses of the cell, which leads to synergistic effects with bactericidal antibiotics. Reprinted with permission from reference [53] © Springer Nature Limited 2021.

Many of the PSMs used in antibacterial therapy have aromatic or heterocyclic ring structural components, with one example being a vast range of monoterpenes in *Melaleuca alternifolia* essential oil [89]. These structural components are suggested to play an important role in the disruption of the functional and structural integrity of cell membranes by preferentially partitioning themselves into membranes, confirmed experimentally by the leakage of ions, inhibition of respiration, and changes in cellular morphology in cells exposed to such PSMs [89]. However, it has been suggested that exposure of cells to PSMs containing these motifs in their backbone may upregulate the transcription of genes responsible for the induction of a particular efflux system, rendering these cells more efficient at expelling other antimicrobials e.g., conventional antibiotics, and thus contributing to antibiotic tolerance or resilience. The presence of CAP-generated active species, in particular ROS, may subject the cell to the level of environmental stress that would promote the efflux pump expression process [90,91,92]. For instance, MexXY-OprM and MexEF-OprN antibiotic efflux systems have been shown to be induced by oxidative and nitrosative stress, respectively, with cells expressing these efflux pump systems being selected for under in vitro stress conditions [93,94,95]. Furthermore, the presence of a multitude of CAP-modified PSM derivatives that vary in their structure, hydrophobicity, and ionizability may additionally contribute to the overexpression of efflux pumps, possibly affecting the transport of PSMs as well as transient resistance to antibiotics (Figure 6). 

### 4.2. Upregulation of Defense Mechanisms

Exposure to certain types of PSMs have been demonstrated to induce oxidative stress response genes. For example, in *E. coli*, indole, a PSM that also plays an important role in the signaling within and across bacterial species [96,97], has been shown to not only upregulate the expression of antibiotic efflux systems [98], but also of OxyR-regulated genes related to the synthesis of alkyl hydroperoxide reductases, thioredoxin reductase and the DNA-binding protein Dps [99,100,101], both positively contributing to the emergence of persisters able to survive the treatment by fluoroquinolone, aminoglycoside, and β-lactam [99] antibiotic families. It should be noted that when exposed to a hydroxyl radical or another potent oxidant, such as those readily produced by CAP treatment, indole can be one-electron reduced to a radical, adding to endogenous ROS and potentially promoting OxyR activation [102]. When dissolved in membrane lipids, indole can induce changes in the membrane molecular arrangement and consequently enable redox-cycling isoprenoid quinones to come into direct contact with dioxygen, generating superoxides [101]. Hence, it may be necessary to consider the effect of repeated exposure of bacterial populations to PSMs in terms of the resilience the treated cells may develop to CAP-generated ROS species.

Similarly, exposure to CAP-generated chemical and physical effects can upregulate the synthesis of genes responsible for cell survival [103,104]. As discussed earlier in this paper, CAP produces a variety of potentially biologically active effects, including highly reactive radicals and ions [105] (many of which also occur naturally in cells where they play an important role in, e.g., signaling and metabolism), mild heating, electric fields, physical agitation, and dehydration due to air flow, shockwave effects, and many others. Jointly, these effects mimic naturally occurring environmental stresses to which microorganisms have evolved effective defense response mechanisms, and application of these stresses simultaneously provides a pathway to activation of these defenses [106]. Importantly, exposure to one of these stresses, e.g., mild heating, may afford protection against another, seemingly unrelated stress, e.g., ethanol toxicity [107] or excessive levels of salts [108]. Such cross protection is a result of the upregulation of expression of general survival genes induced by non-lethal agitation, including CAP agitation [109], and manifested through the synthesis of osmolytes such as glycerol to stabilize aggregated proteins and prevent their misfolding, trehalose to protect cells from desiccation, heat shock proteins (HSPs) that help cells maintain their proteostasis, and radical oxygen scavengers that detoxify exogenous and endogenous ROS, as well as through enhanced chaperone and proton pumping activity, and modulation of redox control, carbon/nitrogen balance, and ion and water transport [110,111,112,113,114]. Once upregulated and accumulated, these molecules and processes allow cells to rapidly respond to reapplication of a stress factor through repair of any macromolecular damage, as well as by transitioning to a state of living where further damage is minimized. 

### 4.3. Consumption of ROS

Earlier in the paper, the ability of some PSMs to increase ROS production in cells and in doing so enhance ROS-related CAP toxicity has already been discussed. Being redox active, PSMs such as 2-heptyl-3-hydroxy-4-quinolone can reduce both free radicals and metal ions, with the latter reaction promoting ROS generation via the Fenton reaction [115]. Given that CAP treatment can temporarily increase the permeability of the membrane and in doing so allows more effective transport of PSMs into cells, the intracellular generation of ROS mediated by PSMs may also be amplified [104,116]. Having said that, not all PSMs lead to oxidative stress generation, with many representatives of terpene, tannin, flavonoid, and saponin families showing different levels of antioxidant activity. These PSMs may interact with CAP-generated ROS directly, effectively scavenging ROS from the environment. It should be noted that in addition to scavenging CAP-generated ROS, PSMs, such as carotenoids with highly conjugated polyene backbones, may remove macrophage- and neutrophil-generated ROS, thereby reducing the efficacy of host immune response. CAP treatment has previously been shown to stimulate host immunity, so it is possible that, at least in part, the efficacy of CAP treatment of infected wounds would be attributable to increased host immune cell activity [117]. Importantly, the scavenging activity may take place both extracellularly as well as intracellularly, thereby dampening oxidative stress that may have arisen intracellularly in response to CAP and PSM treatment. Clearly, there is a need to better understand the interplay between potentiation and attenuation of CAP and PSM efficacy related to redox balance; however, this is not a trivial task given the complexity and diversity of possible mechanisms and cascades. For example, endogenous hydrogen sulfide produced by microbial cells in response to oxidative stress can reduce oxidative stress by sequestering free iron that is necessary for the genotoxic Fenton reaction [118], while at the same time stimulating the activity of catalase and superoxide dismutase. The complexity only increases when one considers real life scenarios of polymicrobial communities, where interspecies protection has been widely reported, host diseases, and medication to which the patient and hence the infectious agents may have been exposed to, e.g., conventional antibiotics. For instance, patients with cystic fibrosis have lung environments rich in bioavailable ferrous iron (Fe(II)). *Pseudomonas aeruginosa* tends to successfully colonize the lungs and show significant resistance to antibiotics such as aminoglycosides and polymyxins, attributed to its ability to sense Fe(II) using a BqsRS signal transduction system, activating transcription of genes associated with polyamine transport and biosynthesis [119]. *P. aeruginosa* has also been demonstrated in vitro to produce signaling molecules that positively contribute to the resistance of *Candida albicans* to fluconazole.

## 5. Conclusions

The need for alternative antimicrobial treatment methods is becoming crucial. CAP can enhance the antimicrobial efficiency of PSMs by altering their chemical and physical properties, enhancing their biochemical activity, and promoting their transport into cells. In parallel, PSMs can contribute to ROS generation and interfere with damage repair processes. At the same, there is strong potential for antagonistic effects, particularly with respect to ROS scavenging and efflux pump activation. Further research is needed to understand the exact antimicrobial mechanism of CAP and PSMs and their co-application, with specific focus on possible synergistic and antagonistic interplay between reactive species in CAPs and bioactive molecules in PSMs in order to fully exploit the potential of this co-therapy against multidrug-resistant bacteria. However, it is also important to note that both CAP and PSMs exhibit potential for toxic effects, depending on specific conditions and applications. CAP, can induce cytotoxicity, DNA damage, and oxidative stress in cells, while PSMs can be toxic when consumed excessively, causing allergies in sensitive individuals. Careful consideration of dose-dependent effects and context is crucial, as both CAP and plant secondary metabolites have potential benefits when used intentionally and with awareness of their associated risks.
